# Inhibitory DAMPs in immunogenic cell death and its clinical implications

**DOI:** 10.15698/cst2021.04.247

**Published:** 2021-03-22

**Authors:** Kazukuni Hayashi, Fotis Nikolos, Keith S. Chan

**Affiliations:** 1 Department of Pathology and Laboratory Medicine, Cedars-Sinai Medical Center, Los Angeles, CA, 90048, USA.; 2 Graduate Program in Translational Biology and Molecular Medicine, Baylor College of Medicine, Houston, TX, 77030, USA.; 3 Samuel Oschin Cancer Center, Cedars-Sinai Medical Center, Los Angeles, CA, 90048, USA.

**Keywords:** gemcitabine, chemotherapy, damage-associate molecular pattern, immune-checkpoint blockade therapy, immunogenic cell death, prostaglandin E2

## Abstract

Dying (or dead) cells are increasingly recognized to impose significant biological influence within their tissues of residence—exerting paracrine effects through proteins and metabolites that are expressed or secreted during cellular demise. For example, certain molecules function as potent mitogens, promoting the repopulation of neighboring epithelial cells. And other myriad of factors—classified as damage-associated molecular patterns (DAMPs)—function as “find me” (attractant), “eat me” (engulfment), or “danger” (activation) signals for recruiting and activating effector immune cells (e.g., dendritic cells) to initiate inflammation. Since the discovery of immunogenic cell death (ICD), the current dogma posits DAMPs as immunological adjuvants for innate immune cell mobilization and activation, which ultimately leads to the antitumoral cross-priming of CD8^+^ T cells. However, what is currently unknown is how these immunostimulatory DAMPs are counteracted to avoid immune-overactivation. Our recent work builds on these fundamentals and introduces prostaglandin E_2_ (PGE_2_) as an ‘inhibitory' DAMP—a new variable to the ICD equation. Prostaglandin E_2_ functions as an immunosuppressive counterpoise of adjuvant DAMPs; and thus, mechanistically precludes ICD. Furthermore, the long-debated immunogenicity of gemcitabine chemotherapy was revealed to be contingent on inhibitory DAMP blockade and not due to its inability to promote DAMP expression (i.e., calreticulin) as previously reported. These findings were intriguing. First, despite the presence of gemcitabine-induced hallmark DAMPs, the inhibitory DAMP (i.e., PGE_2_) was sufficient to hinder the ICD-induced antitumoral immune response (Fig. 1a). And second, rather than pharmacologically substantiating immunostimulatory DAMPs as conventionally approached, the mitigation of the inhibitory DAMP—tipping the immunostimulatory and inhibitory DAMP balance in favor of immunostimulatory DAMPs—was sufficient to render the cell death immunogenic and converted gemcitabine into an ICD-inducing therapy (Fig. 1b). In this microreview, we extrapolate our findings and implicate the value of inhibitory DAMP(s) in drug discovery, its use for clinical prognosis, and as target(s) for therapeutic intervention.

## INHIBITORY DAMP OR INHIBITORY DAMPS?

How are immunostimulatory DAMPs shut off? Under normal physiological conditions, the co-stimulatory molecules, CD80/86, on antigen presenting cells bind to the co-activating signal, CD28, on T cells to bolster their activation. In contrast, CD80/86 can also bind to the corresponding co-inhibitory signal, CTLA4, which functions as a competitive buffer to prevent uncontrolled T cell over-activation (i.e., autoimmunity). Thus, it seems plausible that there are co-inhibitory mechanism(s) set in place to prevent the overt DAMP-driven immune activation. Currently, only a few DAMPs have known antagonists; of such, interleukin-1 (IL-1) and its receptor antagonist (IL-1Ra). The discovery of PGE_2_ as an inhibitory DAMP (iDAMPs) provides a rheostat-like mechanism that tightly regulates inflammation during ICD. Such finding begs the conceptual question, are there additional iDAMPs to fine-tune inflammation? Rather than a system that is solely dependent on the hallmark, adjuvant DAMP signaling, it is rational to think of ICD as a tug-of-war (or a delicate balance) between both immunostimulatory and inhibitory DAMPs. Here, we provocatively hypothesize that newer iDAMPs could be discovered within the next decade, and these newer findings will have a profound impact on the conceptualization of ICD and other physiological conditions.

## iDAMP(S) IN DRUG DISCOVERY AND CLINICAL PROGNOSIS

Currently, only a handful of drugs are considered as bonafide ICD-inducers. The elegant integration of DAMP biosensor cell lines that measures fluorescence to distinguish *i)* the cell surface translocation of CRT from the endoplasmic reticulum and *ii)* the extracellular release of HMGB1 and ATP, has paved the path to systematically identify novel ICD-inducing drug candidates. How would iDAMP(s) conceptually impact ICD-drug discovery? Currently, amongst a panel of candidate drugs that molecularly promote hallmark DAMPs, only a few are capable of inducing ICD *in vivo*. Under these circumstances, are iDAMP(s) a cause for the low ICD-drug turnout? The incorporation of iDAMP(s) into the drug discovery pipeline may better assess a drug's ICD-inducing potential. Anecdotally, PGE_2_ release was approximately three-folds lower in mitoxantrone-treated (a known ICD-inducer) murine bladder cancer cells, when compared to the gemcitabine-treated cells *in vitro*. Such findings beg the question of whether bonafide ICD-inducers promote less iDAMP release in general? If so, what are the mechanisms? It is possible that anthracyclines (i.e., topoisomerase inhibitors) preclude COX-2 protein synthesis by preventing chromatin decondensation; thus, mitigating RNA translation. RT-qPCR-based assays can easily address this question. Can other ICD-inducers also halt COX-2 mRNA translation by different mechanisms? Further studies are required to dissect these underlying mechanism(s) that regulate iDAMPs during drug-induced cell death. In the meantime, novel drugs that promote hallmark DAMPs, but can also concurrently prevent iDAMP biosynthesis may pose as a lucrative class of ICD-inducers for clinical translation.

The clinical prognostic value of DAMPs remains unresolved. On one hand, calreticulin expression—in the context of chemotherapeutic treatment—in acute myeloid leukemia (n=20), colorectal cancer (n=68), neuroblastoma (n=68), and ovarian cancer (n=22) is favorable. While in bladder cancer (n=30), mantle cell lymphoma (n=92), and neuroblastoma (n=251), calreticulin levels correlate with poor overall survival. Similarly, HMGB1 levels in breast cancer (n=232) positively correlate with favorable prognosis, while in pancreatic cancer (n=78), high HMGB1 is unfavorable. Do these mixed results imply additional iDAMP to be considered as biomarkers? Perhaps, a panel of both stimulatory and inhibitory DAMPs would be appropriate for future clinical studies to resolve their prognostic capacity.

## iDAMP BLOCKADE IN CHEMO- AND CHEMOIMMUNOTHERAPEUTIC SETTING

**Figure 1:**
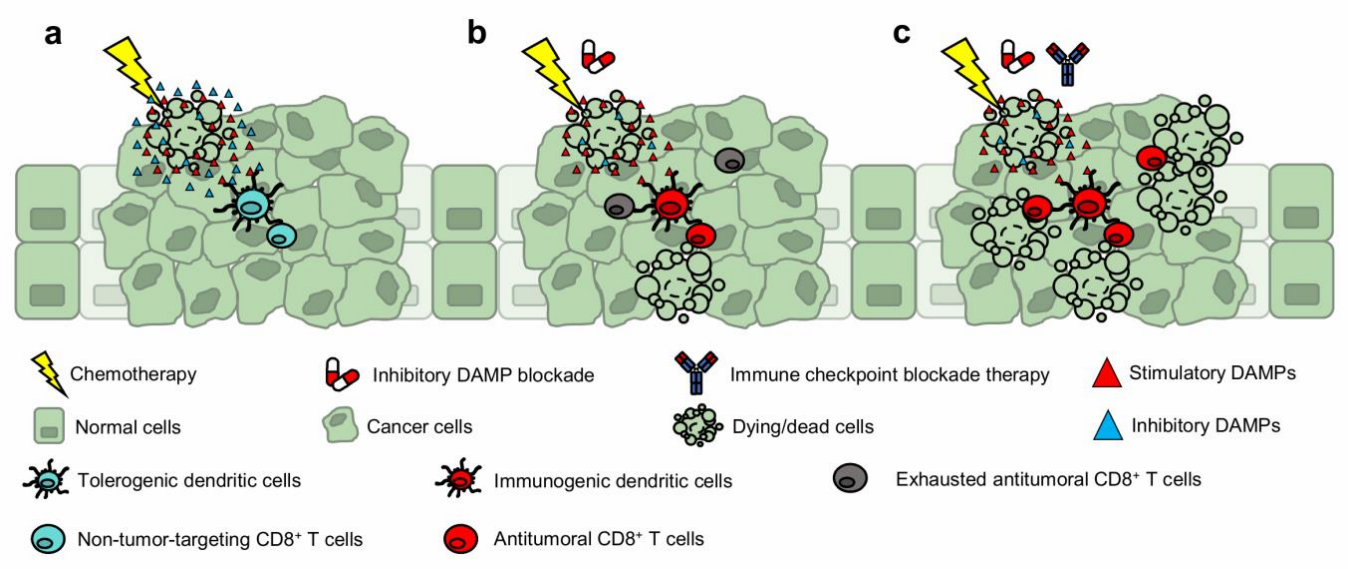
FIGURE 1: Blockade of inhibitory DAMP convert gemcitabine into an ICD-inducer that primes a durable CD8^+^ T cell response. (**a**) Gemcitabine as a single chemotherapy induces tolerogenicity. (**b**) When gemcitabine is complemented with iDAMP blockade, an antitumoral immune response is elicited. (**c**) iDAMP blockade is expected to augment chemotherapy and immune checkpoint blockade therapies.

Conventional strategies utilize adjuvant drugs that substantiate immunostimulatory DAMP expression/release to convert non-ICD-inducers into ICD-inducing drugs. Our current study revealed an alternative approach; inhibitory DAMP blockade converted gemcitabine (a non-ICD-inducer) into an ICD-inducing chemotherapy. By mitigating iDAMP release from dying cancer cells, bone marrow-derived dendritic cells displayed an immunogenic maturation phenotype and vaccine-draining lymph node CD8^+^ T cells were polarized towards a type-I phenotype. However, this strategy was in the context of a vaccination assay. To advance our findings into human clinical trials, we are currently implementing pharmacological iDAMP blockade with gemcitabine-based chemotherapy treatment in preclinical model(s). To generalize the applicability of this strategy, we are evaluating other tumor types (e.g., pancreatic and lung) using the same therapeutic strategy. We hypothesize that concurrent iDAMP blockade during gemcitabine-based chemotherapy treatment will promote a type-I T cell polarizing tumor immune microenvironment, resulting in the selective expansion of tumor-targeting cytotoxic CD8^+^ T cells. Furthermore, we speculate the sequential administration of iDAMP blockade plus chemotherapy followed by immune-checkpoint blockade therapy (ICBT) will prime a durable T cell response, which could increase the spectrum of patient response toward ICBT (**Fig. 1c**). Our rationalization is supported by the works of others, where the combination of ICD-inducing chemotherapy plus ICBT displayed better therapeutic efficacy than chemotherapy or ICBT alone in both pre-clinical and clinical settings. Our finding is timely, since a recent Phase 3 clinical trial combining chemotherapy with ICBT in metastatic bladder cancer (IMvigor130) failed to meet its primary endpoint. Our results, together with those from others, provide a strong rationale to evaluate celecoxib/EP antagonists in combination with chemotherapy and ICBT. We highly anticipate future preclinical and clinical studies to evaluate the efficacy of iDAMP blockade in combination with ICB-based therapies and foresee these discoveries to benefit patients across all cancer types.

In conclusion, we hope that our study will spark the future identification of novel iDAMPs. And that these newer findings will initiate the integration of iDAMPs for assessing ICD in both basic research and clinical settings.

